# Cardiac arrest in children

**DOI:** 10.4103/0974-2700.66528

**Published:** 2010

**Authors:** Erika E Tress, Patrick M Kochanek, Richard A Saladino, Mioara D Manole

**Affiliations:** University of Pittsburgh School of Medicine, Departments of Pediatrics and Critical Care Medicine, 3434 Fifth Avenue, Pittsburgh, PA, 152 60, USA

**Keywords:** Cardiac arrest, cardiopulmonary resuscitation, emergency medicine, pediatrics

## Abstract

Major advances in the field of pediatric cardiac arrest (CA) were made during the last decade, starting with the publication of pediatric Utstein guidelines, the 2005 recommendations by the International Liaison Committee on Resuscitation, and culminating in multicenter collaborations. The epidemiology and pathophysiology of in-hospital and out-of-hospital CA are now well described. Four phases of CA are described and the term “post-cardiac arrest syndrome” has been proposed, along with treatment goals for each of its four phases: immediate post-arrest, early post-arrest, intermediate and recovery phase. Hypothermia is recommended to be considered as a therapy for post-CA syndrome in comatose patients after CA, and large multicenter prospective studies are underway. We reviewed landmark articles related to pediatric CA published during the last decade. We present the current knowledge of epidemiology, pathophysiology and treatment of CA relevant to pre-hospital and acute care health practitioners.

## INTRODUCTION

Pediatric cardiopulmonary arrest is a unique entity, distinct from adult cardiac arrest (CA) in etiology, early pathophysiology and characteristics of the neuronal milieu affected by this disease. CA in children is the result of asphyxia in a majority of the cases. Children have increased cerebral blood flow and higher metabolic needs as compared with adults, and undergo neuronal maturation and synaptogenesis at the time of the insult.

CA is defined as “cessation of cardiac mechanical activity, determined by the inability to palpate a central pulse, unresponsiveness and apnea.” This definition was proposed in the pediatric Utstein guidelines in 1995, the first pediatric guidelines that established definitions and templates for reporting of events surrounding CA.[1] This consensus statement highlighted a global emphasis on improving our knowledge in the field of pediatric CA and marked the start of a period of significant progress in this field. In our review, we will present the epidemiology, pathophysiology and current treatment of CA relevant to the pre-hospital and acute care settings.

## EPIDEMIOLOGY

In the United States, approximately 16,000 pediatric patients suffer CA each year. Younger patients, specifically younger than 1 year of age, comprise the majority of pediatric patients with CA, and males are affected in a slightly higher proportion (62%). A collective review of pediatric cases of CA published a decade ago reported a survival rate to discharge of 13%, with good neurologic outcome in 62% of these patients.[2] This early study in pediatric CA identified that patients who sustain a CA in the hospital setting (in-hospital CA) have better survival as compared with patients with CA out of the hospital (out-of-hospital CA) (24 vs. 8.4%, respectively).

A more recent large cohort study confirmed this observed difference in survival and concluded that patients with in-hospital and out-of-hospital CA must be treated as distinct populations.[3] Given current advances in the field and collaborative efforts among medical centers, there are recent prospective data that define each of these unique populations.

### Out-of-hospital pediatric CA

The incidence of out-of-hospital CA is between 7.5 and 11.2 patients per 100,000 person-years.[4–6] A North American prospective multicenter study determined that the incidence of pediatric CA is greater in infants versus children and adolescents (72.7 vs. 3.7 vs. 6.3 per 100,000 person-years). In that study, the overall survival was 6.4%, lower in infants compared with children and adolescents (3.3 vs. 9.1 and 8.9%).[4] A greater survival rate is seen in pediatric patients with witnessed CA versus unwitnessed CA (13.1% vs. 4.6%), in patients receiving by-stander cardiopulmonary resuscitation (CPR) (9.4 vs. 4.7%) and in patients with return of spontaneous circulation (ROSC) in the field versus patients transported in CA (37 vs. 10%).[4,7] Initial rhythm documented by first responders is asystole or pulseless electrical activity in 82-84% of the patients and ventricular fibrillation in 7–10% of the patients.[3,4,8]

### In-hospital pediatric CA

In-hospital CA occurs in 2–6% of the patients admitted to a pediatric intensive care unit. As expected, a majority (71–88%) of the patients with in-hospital CA have chronic pre-existent conditions, most commonly pulmonary, cardiac, gastrointestinal, neurologic and oncologic.[3,9] Etiology of CA remains similar to that of the out-of-hospital population, asphyxia being most common, followed closely by circulatory shock.[9,10] ROSC is seen in a majority of the patients (64% in one study) and survival to discharge is reported at 16%.[9] The first documented rhythms in a majority of pediatric patients with in-hospital CA are asystole and bradycardia. Ventricular fibrillation or pulseless ventricular tachycardia is reported in 10–14% of the patients with in-hospital CA.[3,11] Improved survival for patients with in-hospital CA in spite of a higher proportion of co-morbidities is probably related to a shorter “no flow” phase secondary to the immediate initiation of CPR.

## PHASES OF CA: PATHOPHYSIOLOGY, PREVENTION AND INITIAL TREATMENT

The are four phases of CA: pre-arrest, no-flow, low flow and post-resuscitation, with distinct pathology and therapy.[10]

The pre-arrest phase consists of events leading to CA. These events include environmental risk factors and pediatric pathologic conditions. The goal of this phase is to identify and treat precipitating factors for CA. In the out-of hospital setting, this is achieved by pediatric caregiver education (i.e., anticipatory guidance) as demonstrated by the reduction of SIDS after the introduction of the “Back to sleep” campaign and reduction of drowning after implementation of swimming safety education.[12] In the pre-hospital and hospital settings, this objective is achieved by the education of providers in the recognition of pediatric pathology leading to CA: respiratory and cardiovascular failure. Asphyxial CA is characterized by a progressive clinical decline in cardiac function initiated by hypoxemia, hypercarbia, acidosis, hypotension and subsequent cessation of cardiac activity. Reversal of respiratory failure during this period of hypoxemic hypoperfusion results in successful prevention of CA.[13] Likewise, improved patient outcomes are seen with early treatment of shock.[14]

The no-flow phase represents untreated CA prior to recognition by a lay bystander in the community or a medical provider in the hospital setting. A majority of out-of hospital pediatric CA are not witnessed, and thus the no-flow phase is longer and cannot be precisely timed. Even in cases of a witnessed CA, bystander CPR is provided to only one-third of the patients. As bystander CPR is associated with increased survival rate (9.4 vs. 4.7%),[4] education of parents and caregivers in the performance of CPR in all communities is imperative. In-hospital CA occurs in settings with closer monitoring and, consequently, the no-flow phase is short. Although this subset of patients with CA have chronic pre-existing conditions more often, survival is better as compared with out-of-hospital CA, probably due to a shorter no-flow phase.[3]

The low-flow phase begins at the initiation of CPR. Chest compressions combined with ventilation provide coronary and cerebral perfusion.[10] The impact of low flow and no-flow states on brain perfusion was demonstrated in an animal study by varying the time of no flow from 1 to 9 min. On initiation of CPR, cerebral blood flow delivered by chest compressions was 20% of the baseline cerebral blood flow if the no-flow time was greater than 1 min, and decreased to zero if the no-flow time was >9 min.[15] Thus, the no-flow time is critical to whether CPR serves to provide adequate cerebral blood flow or simply to generate ROSC. High-quality chest compressions, i.e. hard, fast compressions with full chest recoil, are important. Interruptions in CPR are detrimental.[16] Education and re-education of the professional staff and first responders cannot be overemphasized as studies have shown that CPR delivered by professionals is frequently suboptimal.[17,18] Another important factor during this phase of CA is recognition and treatment of those rhythms that may be treated with defibrillation. Other interventions during this phase can include pharmacologic support, as detailed in the Advanced Life Support section of this article.

The post-resuscitation phase begins with ROSC. While ROSC is the initial therapeutic goal in CA and is a measure of the initial success, post-resuscitation care must be focused on reducing neuronal loss. The presence of a patholophysiologic state after resuscitation was recognized four decades ago by Vladimir Negovsky.[19] If ROSC is achieved quickly after the recognition of CA and initiation of treatment, a pathological post-resuscitation phase may not occur. A majority of patients, however, will experience a varying degree of post-CA syndrome. This term was proposed in 2008 in a consensus statement by the International Liaison Committee on Resuscitation (ILCOR). The phases of post-CA syndrome proposed in their statement are: (a) immediate post-arrest phase, defined as the first 20 min after ROSC, (b) early post-arrest phase, from 20 min to 6-12 h, (c) intermediate phase, 6–12 to 72 h and (d) recovery phase, starting 3 days after ROSC. Post-CA syndrome includes brain injury, myocardial dysfunction and systemic ischemia/reperfusion response.[20]

Post-CA brain injury is a significant cause of morbidity and mortality. It includes impaired cerebrovascular autoregulation, cerebral edema and post-ischemic neurodegeneration. The extent of cerebrovascular disturbances and neurodegeneration is dependent on the duration of CA and on the region of the brain. In a pediatric model of asphyxial CA, cortical hypoperfusion occurred after as little as 9-12 min of ischemia. Early hyperemia was isolated to the subcortical areas and was absent in asphyxias of long duration (12 min).[21] Clinical manifestations of post-CA brain injury include seizures, cognitive dysfunction, myoclonus, signs of stroke, coma, persistent vegetative state and brain death.[20]

Post-CA myocardial dysfunction after pediatric CA is a reversible phenomenon in a vast majority of patients. It is marked by global myocardial hypokinesis (myocardial stunning) and low cardiac output, with normal coronary blood flow. Clinically, it can manifest as hypotension, dysrhythmias and cardiovascular collapse. In swine models, the recovery time of myocardial hypokinesis is between 24 and 48 h.[22] In human studies, cardiac indices in adults resuscitated from CA normalized by 72 h.[23] After pediatric CA, the severity of post-CA myocardial dysfunction is likely less than that after adult CA, similar to data from immature animals.[24]

Systemic ischemia/reperfusion response in CA is secondary to inadequate tissue oxygenation followed by reperfusion. Ischemia begins during the no-flow and low-flow phases of CA and persists even after ROSC due to myocardial dysfunction.[23] Accumulating oxygen debt, defined as the difference between predicted oxygen consumption and actual consumption multiplied by time duration, along with oxygen surplus during reperfusion, causes systemic inflammation, with significant morbidity. Pathologies during this phase include increased coagulation, impaired vasoregulation, adrenal suppression and increased susceptibility to infection.[20] Clinical manifestations include fever, hypotension, hyperglycemia and infection that may precipitate multiorgan failure. This pathophysiology is similar to that seen in the sepsis syndrome.[25]

## TREATMENT OF CA: CPR

Prompt initiation of CPR directly impacts survival and outcomes after pediatric CA. However, the rates of by-stander CPR are low (17–35%).[4,5] By-stander CPR limits the no-flow phase, initiates the low-flow phase and, consequently, increases the coronary and cerebral pressures, increasing the chances of successful resuscitation. The ILCOR published CPR guidelines in 2005.Overall, the emphasis was on performing good-quality CPR in every venue with the recommendations to “push hard, push fast, minimize interruptions; allow full chest recoil, and don’t provide excessive ventilation.”[26]

### Basic life support interventions

Following is a summary of the CPR guidelines proposed in the 2005 ILCOR statement[26]:

#### 1. Activation of emergency medical services

- Non-witnessed CA: “Call fast” approach: the rescuer provides a brief period of CPR before calling for professional help and an AED. The cause of CA is most frequently asphyxia and outcome is improved if by-stander CPR is administered.
- Witnessed sudden collapse: “Call first” approach: the rescuer calls for professional help and an AED followed by initiation of CPR. The cause of the sudden collapse is presumed to be ventricular fibrillation thus the recommendation to “call first” for AED.

#### 2. Ratio for compressions–ventilations

- Lone rescuer provides 30:2 compression–ventilation ratio for all age groups of patients.
- Healthcare provider performing two-person CPR provides 15:2 compression–ventilation ratio for infants and children (of note, ratio of compression–ventilation is 3:1 for neonates).

The optimal compression–ventilation rate for children has not been established. The current recommendations stem from studies in animals proving that interruptions in chest compressions lead to decline in coronary perfusion pressure and from mathematical models supporting compression–ventilation rates higher than 5:1 in children.[16,27]

#### 3. Method of performing chest compressions

- Infant CPR: the two thumb encircling hand technique is preferred for two-rescuer CPR. The two-finger technique is recommended for lone rescuer CPR.
- Child CPR: both one- and two-hand techniques are acceptable.

#### 4. Compression location and depth

The lower third of the sternum should be compressed, with a compression depth of one-third of the anterior–posterior diameter of the chest.

Lay rescuers are instructed to start compressions in an apneic and unresponsive child without checking for a pulse. This recommendation is secondary to evidence that lay rescuers are unable to reliably and timely determine the presence of pulse. Health care providers are instructed to start CPR if they are unable to determine the presence of a pulse in 10 s.

### Advanced life support interventions

Once healthcare providers are involved in resuscitation, high-quality CPR must be continued. The person performing compressions should be rotated every 2 min to minimize fatigue and maximize efficiency in performance of CPR.

A sequence of events follows, starting with the determination of cardiac rhythm, maintenance or establishment of airway and administration of medications.

## TREATMENT OF DISTURBANCES IN CARDIAC RHYTHM

The pediatric dose for manual defibrillation for ventricular fibrillation is 2 J/kg. If this is unsuccessful, the subsequent dose should be 4 J/kg. Automatic defibrillation can be performed with a standard automatic external defibrillator (AED) in children above 8 years or above 25 kg. In children between 1 and 8 years, the use of a pediatric-attenuated AED is preferable if available; however, a standard AED can be used if an attenuated AED is not available. There are insufficient data to make a recommendation for or against the use of AED in infants. Amiodarone can be considered for refractory ventricular fibrillation or ventricular tachycardia in children.[28]

## MAINTENANCE OF AIRWAY AND VENTILLATION

Patency of the airway may be attempted with the jaw-thrust maneuver or chin-lift/head tilt maneuver if trauma is not suspected. Establishment of permanent airway via endotracheal intubation versus ventilating via bag valve mask (BVM) depends on the transport time and the experience of the health care provider. If the transport time is short, BVM is preferred. Ventilation via BVM did not impact survival rates to hospital discharge or neurologic outcome compared with ventilation via a tracheal tube when performed by emergency medicalproviders.[29] The expertise and experience of the healthcare professionals present should be considered. If an advanced airway is placed, it is now a universal recommendation that exhaled CO_2_ monitoring should be used to confirm initial tube placement and to assure maintenance of airway during transport. Cuffed endotracheal tubes are acceptable in the pediatric population and are safe even for children <8 years old, excluding neonates.[30] Other advanced airways such as the laryngeal mask airway or Combitube^®^ have not been studied in children with CA to date. Once an advanced airway has been placed, chest compressions and ventilation occur without interruption. Compressions occur at a rate of 100/min and ventilations should take place at a rate of 8–10/min.

The use of supplemental oxygen during resuscitation is considered standard of care; however, the concentration of oxygen to be delivered is an area of ongoing research. Despite recent promising studies published in animal models[31,32] and newborns resuscitated with room air,[33,34] there are insufficient data in children to recommend for or against a specific FiO_2_. Thus, 100% oxygen is still considered standard of care during resuscitation of children from CA. The FiO_2_ after ROSC should be weaned as tolerated assuring adequate oxygenation.

## MEDICATIONS DURING CPR

The intravenous or intraosseous routes should be used for medications administered during CPR in the pediatric population. The current recommended dose of epinephrine during CA is 0.01 mg/kg of the 1:10,000 concentration. Subsequent intravascular doses of epinephrine are recommended every 3–5 min during resuscitation at the same dose. High-dose epinephrine is no longer recommended due to the potential for worse neurologic outcome.[35] There are insufficient data for the use of other medications during CPR in children.

## POST-RESUSCITATION CARE

After ROSC, the therapeutic focus is to optimize cerebral perfusion and cardiac output. The goals of the immediate and early phases of the post-CA syndrome are to avoid hyperthermia, maintain normotension, normoglycemia and normocarbia and avoid hypoxia.[36] To date, no therapy has been shown to improve outcomes in the pediatric population post-arrest. Hypothermia is to be considered for patients who remain comatose after resuscitation.[28]

Monitoring patients in the post-CA period ensures detection of clinical decline at the earliest time-point possible and minimizes secondary brain injury. This concept is defined by ILCOR as early goal-directed therapy or early hemodynamic optimization.[20] During this phase of recovery, the goal is to maintain a balance between delivery of oxygen systemically and overall oxygen demands. This fine balance can be indirectly monitored through mixed venous oxygen saturation. Monitoring via arterial catheter, pulse-oximetry, continuous electrocardiogram and temperature along with general laboratory studies is essential. Additional monitoring tools include pulmonary arterial catheters and echocardiography to measure cardiac function. It is not uncommon to have significant hemodynamic instability during the post-CA syndrome. Hypotension, dysrhythmias and low cardiac index are to be expected.[23,37] The initial intervention in this situation is the use of intravenous fluid to maintain right-heart filling pressures, with the addition of inotropes and vasopressors. Hemodynamic stabilization will assure optimal cerebral perfusion.

Seizures may occur during the post-CA syndrome. The incidence of seizures is not clearly identified in the pediatric post-CA population, but in adult studies it has been seen in 5–15% of the patients.[20] Prolonged seizures can worsen cerebral injury and hence prompt detection and treatment would be imperative to improving neurologic outcome. Thus, the use of continuous electroencephalographic (EEG) bedside monitoring should be considered as an adjunctive monitoring tool. Indeed, an area of great current interest is advanced neuromonitoring after CA in children. In addition to continuous EEG, more advanced methods are being explored or considered, such as near infrared spectroscopy, brain tissue oxygen monitoring or microdialysis.[38–40] A neurocritical care service specifically devoted to such patients may also be logical.[41]

Early hypothermia is shown to be beneficial in adults and neonates after CA. Data in children is more controversial and research is underway. In adults after CA, mild hypothermia of 32–34°C was shown to improve the neurologic outcome.[42,43] In term neonates after hypoxic ischemic events, therapeutic hypothermia improved neurologic outcome.[44,45] Large, multicenter randomized control trials of therapeutic hypothermia for the pediatric post-CA population are underway. Hypothermia should be considered for pediatric patients that remain comatose after CA. Conversely, hyperthermia (even an increase by 1°C) worsens neurological outcome and should be avoided.[46]

Another method to aid resuscitation care is Extracorporeal Membrane Oxygenation CPR (ECPR). It can also be an adjunct for patients with extreme cardiac instability. ECPR can function as a delivery method for therapeutic hypothermia. Risks include vascular injury, neurologic compromise secondary to ischemia, myocardial stun and infection. A recent metaanalysis of studies in pediatric patients receiving EPCR showed a survival rate of 39.6%. However, complications due, most commonly, to neurologic, renal and infectious causes were also prevalent in 17–27% of the patient population.[47]

Finally, additional insight into the prognosis was provided by the recent report of Rafaat *et al*.[48] In one of the largest pediatric series of drowning victims ever reported, an abnormal admission or early follow-up cranial computed tomography scan was a significantly poor prognostic sign.

## CONCLUSION

The field of pediatric CA benefited from an improved understanding of its epidemiology and pathophysiology during the last decade due to multicenter collaborations. The 2005 ILCOR guidelines emphasize good-quality CPR in the pre-hospital and hospital settings. By-stander CPR improves outcomes but is performed in a minority of patients, identifying a need to improve CPR education in our communities. Treatment of the post-CA syndrome remains mainly supportive; hypothermia should be considered in comatose patients. Advanced neuromonitoring and novel therapies are needed for the next breakthrough.
